# Synergistic Effect of Cu Addition and Pre-Straining on the Natural Aging and Artificial Age-Hardening Behavior of AA6111 Alloy

**DOI:** 10.3390/ma18071635

**Published:** 2025-04-03

**Authors:** Shougang Duan, Yizhe Lu, Aiwen Li, Mingkan Tang, Weilun Chen, Chengyi Huang, Jun Du, Yanping Xu, Yan Yan

**Affiliations:** 1 School of Materials Science and Engineering, South China University of Technology, Guangzhou 510640, China; sgduan0410@163.com (S.D.); l995709739@163.com (Y.L.); 202411085587@mail.scut.edu.cn (A.L.); 19125629185@163.com (M.T.); cwl13924509313@163.com (W.C.); huangcehngyi@algig.cn (C.H.); 2 Algig Aluminum Inc., Nanning 530031, China; xuyanping@algig.cn (Y.X.); yanyan@algig.cn (Y.Y.); 3 Guangxi Key Laboratory of Materials and Processes of Aluminum Alloys, Nanning 530031, China

**Keywords:** Al-Mg-Si-(Cu) alloys, pre-straining, Cu addition, age-hardening response, precipitation

## Abstract

This study systematically investigates the synergistic effects of Cu addition (0–0.7 wt.%) and 2% pre-straining on the artificial aging, natural aging (NA), and bake-hardening response (BHR) of AA6111 alloy. The results reveal that Cu significantly enhances age-hardening capacity and accelerates artificial aging kinetics. The 0.7Cu alloy achieved a 14% higher peak hardness (106.9 HV) than the Cu-free alloy (93.8 HV) while reducing peak aging time by 50% (from 10 h to 5 h). Pre-straining further promoted hardening rates, shortening peak aging times to 2 h for the 0.7Cu alloy. Natural aging (NA) severely suppressed BHR in non-pre-strained alloys, reducing paint baking (PB) increments by 75–77.5% after 14 days. However, the introduction of pre-straining not only reduces the negative effects of NA but also improves the BHR. TEM analysis demonstrated that Cu addition accelerated the precipitation of fine GP zones and β″ phases while pre-straining introduced dislocations that acted as heterogeneous nucleation sites for Q′ phases, refining precipitates and suppressing NA cluster formation. The synergistic combination of 0.7Cu and pre-straining achieved a superior BHR yield strength increment of 68.1 MPa with retained ductility, highlighting its potential for automotive applications requiring balanced formability and post-forming strength.

## 1. Introduction

In recent years, with the acceleration of automotive lightweighting, 6000-series (Al-Mg-Si-Cu) aluminum (Al) alloys have become the preferred material for body-panel components due to their excellent strength–formability balance and good bake-hardening response (BHR) [1,2,3,4]. Among them, the AA6111 alloy sheet has attracted widespread attention from both academia and industry because of its significant strength-enhancement characteristics during the paint baking (PB) process after stamping forming [1,5]. The performance optimization of this alloy is highly dependent on the structural evolution of nanoscale precipitates during artificial aging, and copper (Cu), as a key alloying element, has a decisive impact on the alloy’s aging kinetics and final mechanical properties by changing the precipitation sequence and regulating the type and distribution of precipitates [6,7,8,9]. However, a persistent challenge lies in the adverse effects of NA during room-temperature storage, which leads to premature cluster formation, thereby consuming solute atoms and diminishing the alloy’s capacity for subsequent artificial aging [10,11]. This phenomenon severely compromises BHR, limiting the alloy’s ability to achieve target strength after PB cycles.

In actual industrial production, two methods are commonly used to suppress the negative effects of natural aging (NA): micro-alloying and immediate pre-treatment after solution quenching [12,13,14,15]. Cu is one of the most important micro-alloying elements in Al-Mg-Si alloys [4,6,7,16,17,18,19,20]. The addition of Cu can affect the aging behavior of Al-Mg-Si-based alloys through two mechanisms: firstly, Cu atoms enter the β″ phase to form a more stable L/Q′ phase [21], delaying the over-aging process; secondly, it promotes the precipitation density in the early stage of low-temperature aging by reducing the nucleation barrier of GP zones [6,8]. However, Cu alone cannot effectively reduce the negative effects of NA in alloy sheets. Zandbergen et al. [18] found that alloys with higher Cu content had higher natural aging hardening rates and believed that the addition of Cu would reduce the strain energy of natural aging clusters, thereby releasing more vacancies and promoting the growth of NA clusters. Recent studies have indicated that thermo-mechanical processing, particularly pre-straining (PS), can suppress the NA effect by introducing dislocations that capture quenched vacancies and altering the precipitation path [10,12]. Furthermore, pre-straining can alter the structural types of precipitates. For instance, Weng et al. [22] found that precipitates formed along dislocations can exhibit three characteristics: non-periodic atomic arrangement within the precipitates; segregation of Cu at the precipitate/α (Al) interface; and different orientations presented in a single precipitate.

Studies have shown that the interaction between Cu and dislocations is beneficial to the aging properties of 6xxx-series Al alloys. Ding et al. [23] found that the addition of 0.5 wt.% Cu enhances the effect of pre-straining on improving BHR by promoting the preferential precipitation of the Q′ phase. In contrast, Saito et al. [24] reported that pre-straining did not alter the hardness or precipitate types of a low-Cu (0.1 wt.%) alloy aged at 175 °C for 300 min. These findings indicate that the influence of dislocations on aging behavior is closely related to alloy composition, particularly Cu content. Although the effects of pre-straining have been extensively studied, most research has focused on low-Cu 6xxx alloys (e.g., 6016, 6022). For the high-Cu 6111 alloy, the synergistic interaction mechanisms between Cu and pre-straining remain have not been deeply studied. Elucidating the cooperative effects of Cu and pre-straining on the aging performance of 6xxx series alloys is critical for optimizing their mechanical properties. Therefore, this study systematically investigates the coupled effects of Cu content (0–0.7 wt.%) and 2% pre-straining on the artificial aging behavior, natural aging, and tensile performance of AA6111 alloys. Through hardness testing, tensile characterization, and microstructural analysis (TEM/HRTEM), we elucidate how Cu accelerates precipitation kinetics while pre-straining modulates dislocation-mediated phase nucleation. We provide a mechanistic framework for optimizing 6xxx alloys via composition-process synergy, advancing their applicability in high-performance automotive manufacturing.

## 2. Experimental

**Figure 1:**
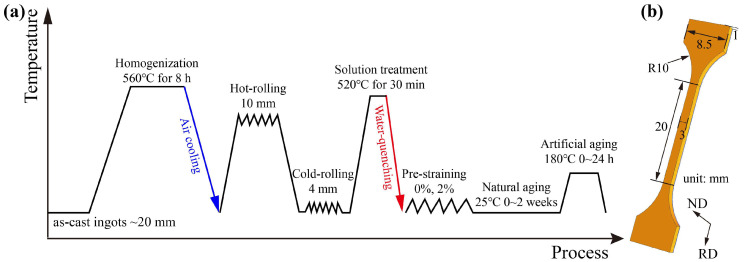
Schematic diagram of (**a**) the process of heat treatment and (**b**) the tensile specimen size.

Table 1 presents the chemical compositions of three experimental Al-Mg-Si-Cu alloys with varying Cu contents (designated as 0Cu, 0.2Cu, and 0.7Cu). The alloys were prepared using industrial pure Al (99.7 wt.%) and master alloys containing Al-10 wt.% Mg, Al-20 wt.% Si, Al-50 wt.% Cu, Al-20 wt.% Fe, and Al-10 wt.% Mn. Precisely weighed raw materials were melted in a graphite crucible using a resistance furnace. To minimize oxidation, the furnace chamber was purged with high-purity argon gas (99.8%) prior to heating, and a continuous argon flow (0.5 L/min) was maintained throughout the melting process. Melting was conducted at 740 ± 10 °C for 40 min with intermittent mechanical stirring to ensure compositional homogeneity. The melt was then poured into a preheated (250 °C) cast steel mold with a rectangular cavity (dimensions: 100 mm × 150 mm × 20 mm) using a tilt-pouring technique. The entire process was completed within an argon-protected environment to prevent gas entrapment and oxide formation. Chemical composition verification was performed through optical emission spectroscopy (HITACHI OE750, HITACHI, Tokyo, Japan) prior to subsequent processing. The as-cast ingots underwent homogenization treatment at 560 °C for 8 h in a forced-air circulation furnace, followed by furnace cooling to an ambient temperature. Surface preparation included mechanical milling to remove 1 mm from each face, eliminating surface oxidation and casting defects. Thermomechanical processing consisted of two stages: hot rolling from 18 mm to 10 mm thickness at 460 °C (reduction rate: 0.8 mm/pass), followed by cold rolling to final 4 mm sheets (total reduction: 60%). Solution treatment was conducted at 520 °C for 30 min in a salt bath, with immediate water quenching to room temperature. Post-quench processing involved two experimental conditions: (1) direct NA for 14 days at room temperature and (2) the application of 2% tensile pre-straining (PS_2_) prior to NA. All specimens subsequently underwent artificial aging at 180 °C for durations ranging from 0 to 24 h. Additionally, a simulated paint bake cycle (180 °C/30 min) was applied to selected samples during natural aging. The complete experimental sequence is schematically illustrated in Figure 1a.

Vickers hardness measurements were conducted using an HVS-1000Z microhardness tester (Caikon, Shanghai, China) under a 500 gf load with 10 s dwell time. Seven indentations per condition were systematically recorded, with the average value and standard deviation calculated to ensure data reliability. Uniaxial tensile testing was performed on room temperature specimens using a Shimadzu AG-X 100 kN universal testing machine (Kyoto, Japan) with a constant crosshead speed corresponding to an initial strain rate of 1.0 × 10^−3^ s^−1^. Three replicate tests per condition were conducted following ASTM E8 standards [25], with the specimen geometry details illustrated in Figure 1b. Microstructural evolution was investigated using a Thermo-Fisher (Waltham, MA, USA) Talos F200X field emission transmission electron microscope (TEM). All TEM and high-resolution TEM (HRTEM) images in this study were acquired along the <100>_Al_ zone axis. TEM samples were prepared by a combination of twin-jet electron polishing with ion thinning. Firstly, the 3 mm diameter disks, which had been mechanically ground to 80 ± 5 μm, were subjected to twin-jet electrolytic thinning using a cryogenic electrolyte (30 vol.% HNO_3_ in methanol, −25 ± 1 °C) under 25.0 ± 2 V potential. Final thinning was achieved using a Gatan 691 Precision Ion Polishing System with low-angle (4 ± 0.5°) 3 keV Ar⁺ bombardment.

## 3. Results

### 3.1. Artificial Age-Hardening Behavior

**Figure 2:**
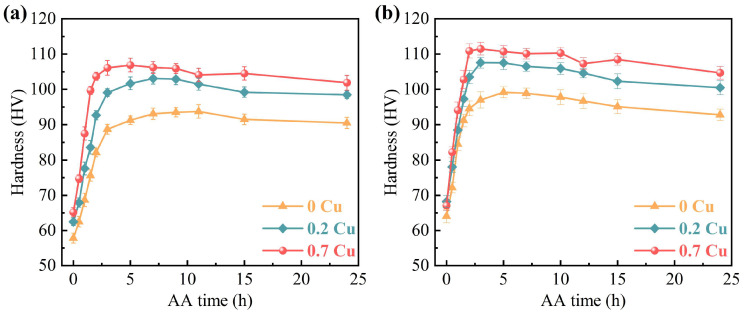
Vickers hardness evolution of the three investigated alloys during 24 h artificial aging at 180 °C, with and without pre-straining. (**a**) Without pre-straining; (**b**) with pre-straining to 2%.

Figure 2 illustrates the artificial aging hardness evolution (180 °C, 0–24 h) of three experimental alloys in both pre-strained and non-pre-strained conditions after two weeks of room-temperature storage. Among the three non-pre-strained alloys, the 0.7Cu alloy exhibited the highest hardness throughout the artificial aging process, while the Cu-free alloy consistently had the lowest hardness (Figure 2a). Additionally, the 0.7Cu alloy consistently exhibited the fastest artificial aging response rate before reaching peak aging. The peak hardness values of the 0.2Cu and 0.7Cu alloys were measured to be 103.1 HV and 106.9 HV, respectively. These values represent increases of 9.9% and 14% compared to the peak hardness of the 0Cu alloy, which registered at 93.8 HV. Regarding the time required to reach peak aging, the 0Cu alloy took 11 h, while the 0.7Cu alloy required only 5 h, signifying a 55% reduction. The peak aging time for the 0.2Cu alloy was also reduced to 7 h. These results demonstrate that Cu addition not only enhances age-hardening capacity but also significantly reduces the time to peak aging.

After a pre-straining of 2%, the three investigated alloys exhibited similar hardening rates in the early artificial aging period (Figure 2b). In comparison to the without pre-straining alloys, the 0.7Cu alloy maintained the highest hardness throughout the artificial aging process, while the 0Cu alloy exhibited the lowest hardness. The peak hardnesses of the 0Cu, 0.2Cu, and 0.7Cu alloys were 99.2 HV, 107.6 HV, and 111.7 HV, respectively, which were 5.4 HV, 4.5 HV, and 4.8 HV higher than those of the non-pre-strained alloys. More importantly, pre-straining significantly reduces the time required to reach peak aging. The peak aging times were reduced to 7 h, 5 h, and 2 h for the 0Cu, 0.2Cu, and 0.7Cu alloys, respectively. This result implies that the introduction of pre-straining effectively promotes the age-hardening behavior of the alloys. In particular, this enhancement is more pronounced in the 0Cu alloy, suggesting potential differences in its response to pre-straining as compared to the 0.2Cu and 0.7Cu alloys.

### 3.2. Natural Aging and Bake Hardening Response

**Figure 3:**
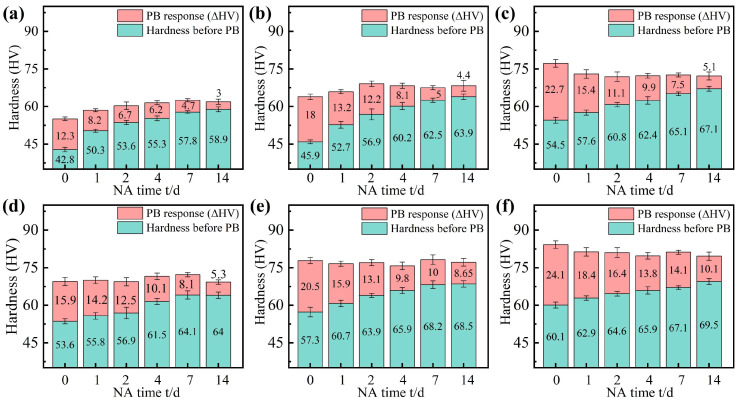
Vickers hardness evolution during two weeks of RT storage and with PB treatment. (**a**–**c**) Non-pre-strained alloys and (**d**–**f**) alloys with 2% pre-straining; (**a**,**d**) the 0Cu alloy, (**b**,**e**) the 0.2Cu alloy, and (**c**,**f**) the 0.7Cu alloy.

Figure 3 demonstrates the NA behavior and PB hardening increments (ΔHV) of AA6111 alloys with varying Cu content (0Cu, 0.2Cu, 0.7Cu) under pre-strained and non-pre-strained conditions during natural aging. The base hardness of the alloys exhibited a progressive enhancement with increasing Cu content. For non-pre-strained samples (Figure 3a–c), a monotonic hardness increase was observed across all alloys as NA duration extended from 0 to 14 days, while the PB increment demonstrated an inverse relationship, decreasing by 75.6% (0Cu), 75.6% (0.2Cu), and 77.5% (0.7Cu), respectively. The 0.7Cu alloy consistently demonstrated superior BHR compared to the Cu-free counterpart throughout the 0–14 days of NA, underscoring the critical role of Cu in retaining bake-hardening potential. Freshly solution-treated samples (without NA) exhibited maximum PB increments, with 0Cu, 0.2Cu, and 0.7Cu alloys achieving 12.3 HV, 18 HV, and 22.7 HV, respectively. After 14-day NA, the hardness of the 0Cu alloy increased from 42.8 HV to 58.9 HV, while the PB increment was only 3 HV (Figure 3a), which was significantly lower than that of the direct PB sample (ΔHV = 12.3 HV). Similarly, the hardness of the 0.2Cu and 0.7Cu alloys increased from 57.3 HV to 68.5 HV and 54.5 HV to 67.1 HV, respectively, while the PB increments were only 4.4 HV and 5.1 HV. Accordingly, although the addition of Cu could enhance the BHR of AA6111 alloy, it is difficult to effectively suppress the negative effects of NA.

The strain hardening resulting from the introduction of 2% pre-straining increased the hardness of all three alloys (Figure 3d–f). For the three pre-strained alloys, the incremental hardness due to (ΔHV_NA_) was reduced after two weeks in natural parking. The ΔHV_NA_ for the 0Cu, 0.2Cu, and 0.7Cu alloys was reduced from 16.1 HV to 10.4 HV, 18 HV to 11.2 HV, and 12.6 HV to 9.4 HV, respectively. Furthermore, the introduction of pre-strain effectively enhances the BHR of the AA6111 alloy. These results indicate increasing the Cu content in combination with pre-straining not only suppresses the negative effects of NA but also enhances the BHR.

### 3.3. Tensile Response

**Figure 4:**
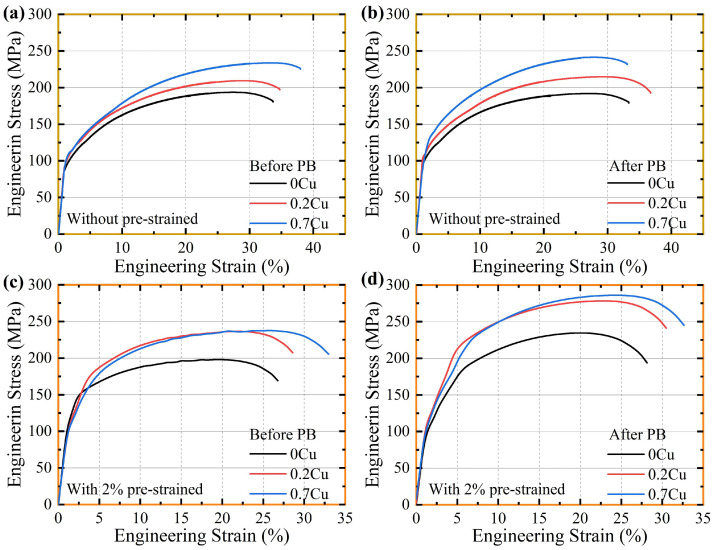
Stress–strain curves (**a**,**c**) before and (**b**,**d**) after PB treatment for three alloys. (**a**,**b**) Non-pre-straining and (**c**,**d**) 2% pre-straining.

Figure 4 presents the engineering tensile stress–strain curves of the three experimental alloys with and without 2% pre-straining, measured before and after PB treatment (180 °C/30 min) following 14-day NA. The quasi-static tensile tests systematically revealed the synergistic effects of pre-straining and Cu alloying on the plastic deformation mechanisms of the AA6111 series Al alloys. The three non-pre-strained alloys exhibited relatively low initial yield strengths (Figure 4a), with limited improvements observed after PB treatment (Figure 4b). Table 2 lists detailed values for yield strength, ultimate tensile strength (UTS), and elongation (El). Quantitative analysis demonstrated that the yield strength increments for 0Cu, 0.2Cu, and 0.7Cu alloys were 12.8 MPa, 14.2 MPa, and 17.7 MPa, respectively, consistent with the hardness results shown in Figure 3. With an increasing Cu content, the alloy strength was enhanced while retaining excellent elongation. After applying 2% pre-straining (Figure 4c,d), all alloys exhibited increased strength with slight reductions in elongation. Notably, pre-straining enhanced the bake-hardening yield strength increment more substantially. The PB-induced yield strength increments for 0Cu, 0.2Cu, and 0.7Cu alloys reached 29.8 MPa, 41.3 MPa, and 68.1 MPa, respectively. In particular, the 0.7Cu alloy not only demonstrated a markedly improved bake-hardening yield strength increment but also maintained satisfactory elongation after pre-straining.

### 3.4. Microstructural Characterization

**Figure 5:**
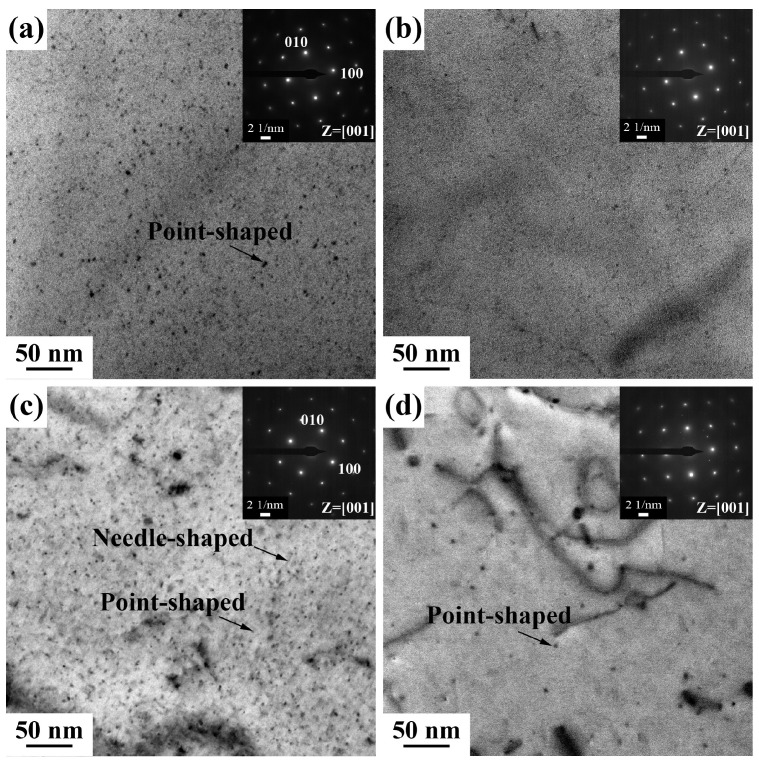
Bright-field TEM images of 0Cu (**a**,**b**) and 0.7Cu (**c**,**d**) alloys after PB without pre-straining. (**a**,**c**) Without NA; (**b**,**d**) underwent 14 days of NA.

**Figure 6:**
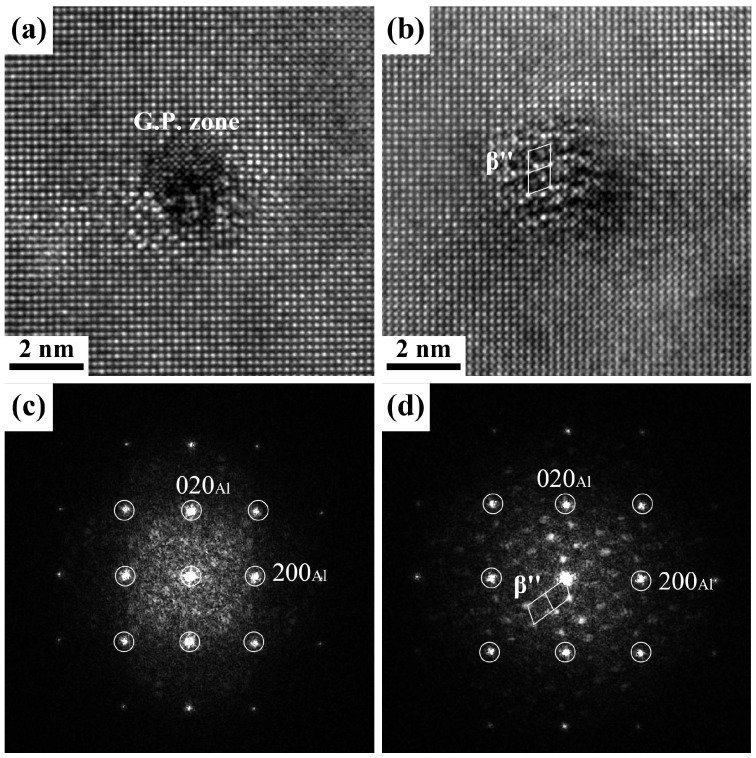
HRTEM images and corresponding FFT patterns of the dot-shaped precipitates in the non-pre-strained alloys. (**a**,**c**) GP zone from 0Cu alloy (0 d); (**b**,**d**) β″ phase from 0.7Cu alloy (14 d).

Figure 5 shows the bright-field (BF) TEM images of the 0Cu alloy and 0.7Cu alloy without pre-straining, both without NA and after 14 days of NA, followed by aging at 180 °C for 30 min. All TEM images were taken along the <100>_Al_ zone axis. For the 0Cu alloy, in the absence of NA, there are a few needle-like precipitates and uniformly distributed fine dot-like precipitates (Figure 5a). Following 14 days of NA, both the size reduction in needle-shaped precipitates and the marked decrease in density of dot-like precipitates indicate intensified detrimental effects of NA, leading to a diminished BHR response in the alloy (Figure 5a). High-resolution TEM (HRTEM) imaging and the corresponding Fast Fourier Transform (FFT) analysis in Figure 6 confirm that the smaller dot-like precipitates correspond to GP zones, while larger ones are identified as β″ phase. In the case of the 0.7Cu alloy (Figure 5c,d), a significantly higher density of dot-like precipitates is uniformly distributed within the Al matrix before NA compared to the Cu-free counterpart, demonstrating that Cu addition accelerates the precipitation of aging–strengthening phases. This aligns with the hardness evolution trends shown in Figure 3. Notably, after 14 days of NA, the post-PB precipitate density in the 0.7Cu alloy also decreases, further confirming that Cu incorporation alone cannot counteract the negative effects induced by NA.

**Figure 7:**
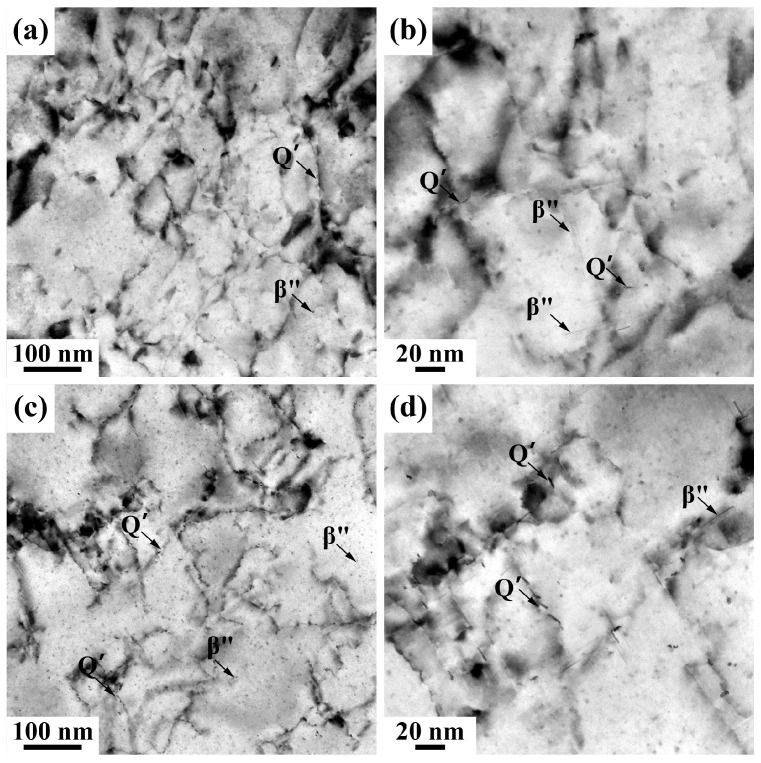
Bright-field TEM images of 0.7Cu alloy with 2% pre-straining subjected to PB treatment after different times of NA. (**a**,**b**) Without previous NA; (**c**,**d**) NA 14 days.

**Figure 8:**
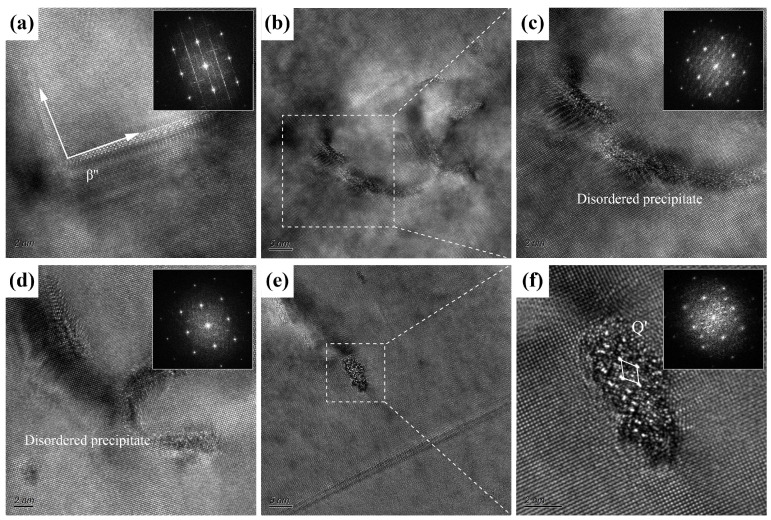
HRTEM images of typical precipitates in 0.7Cu alloy with 2% pre-straining. (**a**) Mutually perpendicular β″ phase; (**b**–**d**) precipitates along dislocation lines; (**e**,**f**) Q′ phase.

To further investigate the effect of pre-straining on the precipitation behavior of the alloy, TEM and HRTEM characterizations were conducted on the 0.7Cu alloy subjected to 2% pre-straining, as shown in Figure 7 and Figure 8. As shown in Figure 7a, a high density of dislocations introduced by pre-straining was observed in the low-magnification BF-TEM image, with numerous precipitates distributed throughout the α-Al matrix. The high-magnification BF-TEM image in Figure 7b revealed that dark dot-shaped precipitates and light needle-shaped precipitates are predominantly distributed near dislocations and within the α-Al matrix. The needle-shaped precipitates correspond to the primary strengthening phase β″, while most dot-shaped precipitates consist of GP zones and the β″ phase, along with a small amount of Q′ phase associated with high Cu content. After 14 days of NA followed by PB treatment, as shown in Figure 7c,d, the 0.7Cu alloy matrix still retained numerous dot-shaped and needle-shaped phases. This indicates that pre-straining significantly inhibited the negative effects of NA. Compared with non-pre-strained samples (Figure 5), the needle-shaped precipitates exhibited greater length and higher density, confirming that pre-straining not only effectively suppresses NA’s detrimental effects but also enhances the alloy’s BHR capability. Following pre-straining, the precipitates growing along dislocation lines displayed continuous, elongated characteristics. Furthermore, these precipitates exhibited disordered crystal structures, as supported by the HRTEM image and corresponding FFT pattern shown in Figure 8.

## 4. Discussion

For 6xxx series Al alloy sheets used in automobiles, BHR and resistance to NA effects are two critical performance indicators [26,27,28]. Superior NA resistance effectively reduces the difficulty of stamping processes for structural components, whereas an enhanced BHR improves the strength of final structural parts [29]. In this study, we investigated the effect of Cu addition on the artificial aging response of the AA6111 alloy. The results demonstrated that increasing the Cu content significantly accelerated the hardening rate and shortened the time to peak aging (Figure 2a). During natural aging, Cu atoms are incorporated into the Mg-Si co-clusters and form Mg-Si-Cu co-clusters [30], thereby increasing the (Mg + Cu)/Si ratio and promoting the formation of Clusters (2) [31,32]. Additionally, prior studies suggest that Cu addition lowers the activation energy of precipitate nucleation [33]. Consequently, during subsequent artificial aging, Cu addition effectively promotes the direct transformation of more Clusters (2) into GP zones and β″ and Q′ phases, resulting in accelerated aging kinetics. With an increasing Cu content, the hardness of solution-treated alloys increases from 57.8 HV for 0Cu to 65.2 HV for 0.7Cu, attributed to solid solution strengthening caused by Cu dissolution in the α-Al matrix. The peak-aged hardness increases from 93.8 HV for 0Cu to 106.9 HV for 0.7Cu, owing to the combined effects of solid solution strengthening and precipitation hardening during subsequent artificial aging. In non-pre-strained alloys, the 0.7Cu alloy exhibited smaller-sized and higher-density precipitates after paint baking (PB) compared to the 0Cu alloy (Figure 5), indicating that Cu-induced microstructure refinement contributes to enhanced precipitation hardening.

While Cu addition alone fails to effectively suppress the adverse effects of natural aging (NA) in alloy sheets, this study further explores the combined influence of pre-straining and high Cu content on NA resistance and artificial aging behavior in the AA6111 alloy. Following 2% pre-straining, the hardness increase during NA in the T4P condition was markedly suppressed (Figure 3). Dislocations introduced by pre-straining trapped quenched vacancies, thereby inhibiting the formation of NA clusters [22,34], with the 0.7Cu alloy exhibiting the most pronounced suppression. During the subsequent PB process, strain fields near dislocations provided heterogeneous nucleation sites for GP zones and enhanced atomic diffusion pathways [35], accelerating the precipitation of strengthening phases. Consistent with prior reports, dislocations in high-Cu alloys promoted the preferential nucleation of the Q′ phase during artificial aging (Figure 7), attributed to the propensity of the Q′ phase to form at defect sites such as dislocations and grain boundaries [6,31]. Notably, precipitates near dislocations exhibited elongated, disordered morphologies, whereas those in dislocation-free regions adopted needle-like (β″) or lath-shaped (Q′) structures [26,36]. This contrast arises from the high interfacial energy and enhanced diffusion near dislocations, which favor directional growth along dislocation lines, while local lattice distortions induce structural disorder [22]. In conclusion, the synergistic effect of Cu and pre-straining enhances the BHR of AA6111 alloy and suppresses the negative effects of NA.

## 5. Conclusions

The findings of this paper provide important insights into the microstructure-property relationships of Cu-modified 6xxx alloys, emphasizing the importance of coupling composition and thermo-mechanical processing in the design of advanced aluminum sheets. The main conclusions are as follows:

(1) Increasing Cu content (0–0.7 wt.%) in AA6111 alloys enhances age-hardening capacity and accelerates artificial aging kinetics. The 0.7Cu alloy exhibited a 14% higher peak hardness (106.9 HV) and 50% shorter peak aging time (5 h vs. 10 h for the Cu-free alloy), attributed to the accelerated precipitation of GP zones, β″, and Q′ phases.
(2) Introducing 2% pre-straining significantly shortened peak aging times (e.g., 2 h for 0.7Cu alloy) and suppressed natural aging (NA) effects by aggregating quenched vacancies and inhibiting NA cluster formation. The pre-strained 0.7Cu alloy achieved a 68.1 MPa yield strength increment after paint baking, outperforming non-pre-strained alloys.
(3) TEM analysis revealed that Cu promotes fine, dense precipitates (GP zones/β″), while pre-straining introduces dislocations that enhance Q′ phase nucleation. Dislocation strain fields facilitated elongated, disordered precipitates along defect sites, improving the BHR and NA resistance.
(4) The synergy of 0.7Cu and pre-straining optimizes AA6111 alloys for automotive applications, balancing a high bake-hardening response (111.7 HV peak hardness) with suppressed NA instability. This combination addresses the trade-off between formability (pre-strain tolerance) and post-forming strength, offering a viable strategy for lightweight component manufacturing.

## Figures and Tables

**Table 1:** Chemical composition of the three investigated alloys (wt.%).

	Mg	Si	Cu	Mn	Fe	Al
0Cu	0.76	0.85	0.01	0.28	0.16	Bal.
0.2Cu	0.72	0.86	0.21	0.28	0.15	Bal.
0.7Cu	0.75	0.86	0.68	0.29	0.16	Bal.

**Table 2:** Tensile properties of the three investigated alloys with and without pre-straining.

Alloys	PS_x_	YS (MPa)		UTS (MPa)		El (%)		BHR (Δ_YS_, MPa)
		Before PB	After PB	Before PB	After PB	Before PB	After PB	
0Cu	PS_0_	91.4	104.2	193.6	191.9	33.6	33.3	12.8
	PS_2_	151.4	181.2	197.8	234.4	26.8	28.2	29.8
0.2Cu	PS_0_	99.6	113.8	209.3	214.6	34.7	36.8	14.2
	PS_2_	176.5	217.8	236.5	278.3	28.6	30.5	41.3
0.7Cu	PS_0_	102.9	120.6	233.8	241.3	37.9	32.9	17.7
	PS_2_	156.8	224.9	237.3	286.1	32.9	32.6	68.1

## Data Availability

The original contributions presented in this study are included in the article. Further inquiries can be directed to the corresponding author.
